# The Impact of Primary Care Practice Models on Indicators of Unplanned Health Care Utilization for Ontario Adults Newly Diagnosed With Chronic Obstructive Pulmonary Disease: A Retrospective Cohort Study

**DOI:** 10.1177/21501319231201080

**Published:** 2023-09-22

**Authors:** Ruixi Sheng, Joan E Tranmer, Christina Godfrey, Thomas Rotter

**Affiliations:** 1 Queen’s University, Kingston, ON, Canada

**Keywords:** emergency visits, health outcomes, primary care, chronic obstructive pulmonary disease, Hospitalization

## Abstract

**Background:**

Chronic obstructive pulmonary disease (COPD) is a highly prevalent chronic disease. Most of the care for this population occurs within the primary care setting; however, the extent to which different primary care practice models influence the outcomes of patients with COPD remains unclear.

**Objective:**

The study aimed to compare and analyze the influence of different primary care practice models on indicators of unplanned health care utilization among newly diagnosed COPD patients in Ontario.

**Design:**

A retrospective cohort study was conducted using health administrative database within the Institute for Clinical Evaluative Sciences. The cohort included persons who were 35 years and older with physician-diagnosed COPD between January 1, 2014 and December 31, 2019. Patients were assigned into 3 practice models: team-based, traditional, and no enrolment. The primary outcomes examined was unplanned health care utilization, specifically emergency department (ED) visits and hospitalizations. To account for excessive zero values, the zero inflated negative binomial (ZINB) models were used to analyze the association between different practice models and unplanned health care utilization.

**Results:**

Among 57,145 individuals who met the inclusion criteria, 55,994 were included in the regression analysis. Of the included participants, 62.8% of patients were in the traditional group, 30.3% were in the team-based group, and 6.9% were in the no enrolment group. Between 2014 and 2019, 70.7% of the cohort had at least 1 all-cause ED visit without hospitalization. The adjusted ZINB models showed no significant difference in risks of experiencing an unplanned health care utilization between the team-based and traditional groups. However, patients in the no enrolment group had a significantly higher risk of ED visit without hospitalization regardless of cause, ED visit with hospitalization regardless of cause, and 30-day readmissions regardless of cause.

**Conclusions:**

Primary care practice models are complex, influenced by remuneration and organizational structures, reinforcing the need for further research to enhance our understanding of primary care reforms. Furthermore, given the growing shortage of primary care providers, patients with COPD and other chronic conditions are particularly vulnerable.

## Introduction

Chronic diseases are long-lasting, typically incurable conditions that impact many individuals worldwide. Chronic diseases account for almost 71% of all deaths^1^ and impose a significant economic burden as they are responsible for 55% of direct and indirect health care costs in Ontario.^2^ Among respiratory diseases, chronic obstructive pulmonary disease (COPD)—a chronic lung disease—is a growing health issue in Canada as it is one of the leading causes for hospitalization.^3,4^ Individuals with COPD are frequent users of the health care system, and have, on average, 3 or more hospitalizations per year with a total length of 30 or more days per year.^3^ Annually, COPD costs the health care system an estimated $1.5 billion dollars.^3^ COPD has a gradual onset, characterized by inflammation and eventual obstruction of the small airways, leading to destruction of lung parenchyma.^5^ Treatment for COPD focuses on stabilization to slow the disease progress and manage symptoms such as chronic cough, sputum production, and persistent dyspnea.^5^ With effective management, COPD exacerbations and consequent hospital admissions and emergency department (ED) visits could be minimized.^6^

COPD is identified as an ambulatory care sensitive condition (ACSC)—meaning potentially unplanned health care utilization can be reduced with comprehensive and accessible outpatient care.^7^ ACSC-related hospitalizations serve as an indirect measure of access to or quality of primary care.^7^ Studies have shown that a higher risk of ACSC-related hospitalizations is associated with ineffective primary care contributed by limited access or inadequate care.^8,9^ Variation in hospital admissions for ACSCs could potentially indicate differences in quality of care among the primary care models, although a certain degree of variation is unavoidable. Similar to hospital admissions for ACSC that are considered “preventable hospitalizations,” many ED visits for ACSCs may be potentially avoidable. Although not all ACSC-related health care encounters are preventable, appropriate, and timely primary care may prevent future exacerbations and improve the quality of life for individuals living with chronic diseases.

Primary care serves as an anchor for patients and families and is well positioned to enable effective care coordination. As the first contact or entry into the health care system, primary care provides the foundation of organizing and activating the necessary health care services required to meet an individual’s health needs. Many patients with COPD are managed predominantly in the primary care setting. In a Canadian survey, most patients with COPD (79%) reported they received regular care from a primary care physician.^10^ Primary care physicians, or family physicians (FPs), play a key role in supporting and facilitating self-management support through continually assessing, monitoring, and managing signs and symptoms. Self-management is especially important for individuals living with chronic diseases as they require life-long education to gain the confidence, knowledge, and skills needed to manage all aspects of the illness.^11,12^ Although patients with COPD can be effectively cared for by their FP, they may also require specialized care to manage complex respiratory symptoms or comorbidities. Respirologists, also known as pulmonologists, are physicians who have specialized training in managing respiratory diseases and are often involved in cases of severe and complex COPD.

Team-based care and care coordination in primary care are concepts widely studied among patients with chronic diseases and are associated with improved disease management.^13-16^ One of the most prominent approaches to quantifying care coordination is by measuring care continuity. Care continuity uses physician claims data to reflect the longitudinal patient-provider relationship by measuring the frequency of visits.^17^ A long-term physician-patient relationship for patients with chronic diseases is thought to improve communication that may lead to more effective management.

Within the province of Ontario, residents have access to a variety of different primary care models with varying organizational and payment structures. In the past, the dominant model was fee-for-service (FFS) in which FPs practiced solo or in groups. Over the past 2 decades, many provinces have undergone primary health care reforms to improve the access and quality of primary care, whether through structural changes (ie, new payment systems, interdisciplinary teams) and/or enhancements in supports (ie, implementation of various patient navigators as observed in most provinces in Canada, such as Nova Scotia, Ontario, and British Columbia).^18^ The common objectives were to increase access to primary care, promote multidisciplinary team-based care, and improve chronic disease management. Between 2001 and 2006, the Ontario government implemented several different primary care models consisting of different reform strategies such as rostering patients into an enrolment model, recommending a minimum number of practitioners within a group, and changes to remuneration. These reforms resulted in changes within the provincial primary care system.

Primary care practice models and physician remuneration serve as important determinants of access to primary care. The term “practice model” refers to organizational difference of models, such as the presence of team-based care, whereas the term “primary care model” is a general term that includes organizational and remuneration differences. Currently in Ontario, there are 3 distinctive types of practice models: 1) the traditional model where physicians practice solo or in groups, 2) models with team-based approaches, and 3) models that provide episodic care. All 3 types of practice models are reimbursed through unique remuneration packages. Although the focus of this study is on practice models, remuneration methods are a key factor that need to be considered. The provincial government relies heavily on changes in physician remuneration, such as variation in incentives and bonuses to improve primary care delivery.^19^ However, relative to the substantial investments on reforming primary care in Ontario, the reforms have not yet produced the expected level of improvement in access or quality in care. Information of funding and practice models in Ontario can be seen in Tables 1 to 6.

**Table 1.** Funding and Practice Models in Ontario.

Model	Definition
Funding (remuneration) models	
Rural and Northern Physician Agreement (RNPGA)	A specialized remuneration model mainly in regions of rural Ontario with salary, incentives, bonuses, shadow-billing premiums, and comprehensive-care capitation as its main payment elements.
Family Health Network (FHN) and Family Health Organization (FHO)	Both are a blend of capitation and salary. FHO differs in the number of services provided.
Family Health Groups (FHG)	An enhanced FFS payment model with rostering (patient enrolment), allowing for incentives that encourage specific health targets.
Comprehensive Care Model (CCM)	Enhanced FFS payment model with additional bonuses and incentives. This method provides higher remuneration while practicing independently.
Primary care practice models	
Traditional Primary Care Models Remuneration methods: • Fee-for-service (FFS) • Enhanced FFS + Incentives (FHGs and CCM) • Blended Capitation (FHNs and FHOs)	The traditional practice model, involving either a solo or a group of general practitioners with no interprofessional aspects. This is further differentiated by the remuneration method. Patient enrolment is required for FHG/FHN/FHO but not for FFS.
Community Health Centre (CHC) Remuneration method: • Salary	One of the oldest practice models, with a focus on population needs with an expanded scope of health promotion and community services. Includes an interdisciplinary approach.
Rural and Northern Physician Group Agreement (RNPGA) Remuneration method: • Complement-based base remuneration plus bonuses and incentives	RNPGA is a specialized model that serves the communities in Northern Ontario. Patient enrolment is required.
Family Health Teams (FHT) Remuneration methods: • Complement-based base remuneration plus bonuses and incentives (FHTs in Northern Ontario) • Blended capitation model (FHN, FHO)	A newer model based on an interprofessional approach that includes a team of family physicians, nurse practitioners, registered nurses, dieticians, social workers, and other health care professionals focused on maintaining a patient’s health and meeting the community’s needs. Only physicians that accept a blended capitation payment method and are members of a FHO or FHN can become an FHT. Patient enrolment is required.
Walk-in Clinics Remuneration method: • FFS	Walk-in clinics offer episodic patient care. Patient enrolment is not necessary.

Source: Information in this table is taken from works by Health Force Ontario,^20^ Liddy et al,^21^ and Sweetman and Buckley.^19^

**Table 2.** Patient Characteristics.

Characteristics	Cohorts			Total combined cohort included in regression analysis	*P*-value^a^	Additional analysis^b^	
	Team-based	Traditional	No enrolment			Other Enrolment	Total combined cohorts
	N (%) *M* ± SD	N (%) *M* ± SD	N (%) *M* ± SD	N (%) *M* ± SD		N (%) *M* ± SD	N (%) *M* ± SD
# of patients	16 960	35 157	3877	55 994		1151	57 145
Sex							
Female	8457 (49.9)	16 773 (47.7)	1670 (43.1)	26 900 (48.0)	<.0001	622 (54.0)	27 522 (48.2)
Male	8503 (50.1)	18 384 (52.3)	2207 (56.9)	29 094 (52.0)		529 (46.0)	29 623 (51.8)
Age group, n (%)							
Mean	62.8 ± 14.0	61.2 ± 14.4	60.1 ± 14.3	61.6 ± 14.3	<.0001	62.7 ± 14.0	61.6 ± 14.3
New immigrant status, n (%)							
Y	587 (3.5)	5151 (14.7)	454 (11.7)	6 192 (11.1)	<.0001	18 (1.6)	6210 (10.9)
Dependency quintile							
Q1 (least)	2035 (12.1)	6843 (19.5)	651 (17.2)	9529 (17.1)	<.0001	35 (3.1)	9564 (16.9)
Q2	2734 (16.3)	6825 (19.5)	716 (18.9)	10 275 (18.5)		68 (6.0)	10 343 (18.2)
Q3	3097 (18.5)	6674 (19.1)	741 (19.5)	10 512 (18.9)		181 (16.0)	10 693 (18.8)
Q4	3476 (20.7)	6707 (19.2)	752 (19.8)	10 935 (19.7)		329 (29.1)	11 264 (19.9)
Q5	5438 (32.4)	7947 (22.7)	934 (24.6)	14 319 (25.8)		518 (45.8)	14 837 (26.2)
Deprivation quintile							
Q1 (least)	2515 (15.0)	6053 (17.3)	497 (13.1)	9065 (16.3)	<.0001	152 (13.4)	9217 (16.2)
Q2	3073 (18.3)	6724 (19.2)	566 (14.9)	10 363 (18.6)		125 (11.1)	10 488 (18.5)
Q3	3460 (20.6)	6677 (19.1)	694 (18.3)	10 831 (19.5)		205 (18.1)	11 036 (19.5)
Q4	3709 (22.1)	7217 (20.6)	860 (22.7)	11 786 (21.2)		292 (25.8)	12 078 (21.3)
Q5	4023 (24.0)	8325 (23.8)	1177 (31.0)	13 525 (24.3)		357 (13.6)	13 882 (24.5)
Ethnic concentration quintile							
Q1 (least)	5771 (34.4)	5967 (17.0)	747 (19.7)	12 485 (22.5)	<.0001	550 (48.6)	13 035 (23.0)
Q2	4445 (26.5)	6343 (18.1)	721 (19.0)	11 509 (20.7)		304 (26.9)	11 813 (20.8)
Q3	3095 (18.4)	6636 (19.0)	700 (18.5)	10 431 (18.8)		182 (16.1)	10 613 (18.7)
Q4	2137 (12.7)	7215 (20.6)	789 (20.8)	10 141 (18.2)		74 (6.5)	10 215 (18.0)
Q5	1332 (7.9)	8835 (25.2)	837 (22.0)	11 004 (19.8)		21 (1.9)	11 025 (19.5)
Instability quintile							
Q1 (least)	1947 (11.6)	5820 (16.6)	409 (10.8)	8176 (14.7)	<.0001	83 (7.3)	8259 (14.6)
Q2	3151 (18.8)	6013 (17.2)	570 (15.0)	9734 (17.5)		193 (17.1)	9927 (17.5)
Q3	3797 (22.6)	6430 (18.4)	670 (17.7)	10 897 (19.6)		272 (24.1)	11 169 (19.7)
Q4	4041 (24.1)	7032 (20.1)	838 (22.1)	11 911 (21.4)		318 (28.1)	12 229 (21.6)
Q5	3844 (22.9)	9701 (27.7)	1307 (34.4)	14 852 (26.7)		265 (23.4)	15 117 (26.7)
ON-Marg score^c^							
Mean	3.1 ± 0.75	3.2 ± 0.79	3.3 ± 0.78	3.1 ± 0.78	<.0001	3.2 ± 0.68	3.1 ± 0.8
Income quintile, n (%)							
1 (lowest)	4219 (24.9)	8545 (23.3)	1244 (32.2)	14 008 (25.1)	<.0001	305 (26.5)	14 313 (25.1)
2	3658 (21.6)	7778 (22.2)	876 (22.7)	12 312 (22.0)		251 (21.8)	12 563 (22.0)
3	3299 (19.5)	6975 (19.9)	686 (17.7)	10 960 (19.6)		227 (19.7)	11 187 (19.6)
4	2996 (17.7)	6153 (17.5)	603 (15.6)	9752 (17.4)		208 (18.1)	9960 (17.5)
5 (highest)	2770 (16.3)	5647 (16.1)	457 (11.8)	8874 (15.9)		159 (13.8)	9033 (15.8)
Lives in a rural area Y/N							
Y	4189 (24.7)	2703 (7.7)	472 (12.2)	7364 (13.2)		257 (22.4)	7621 (13.4)
Rurality^d^ index							
Mean	21.2 ± 22.5	8.1 ± 13.4	10.4 ± 18.2	12.2 ± 18.2	<.0001	30.7 ± 21.7	12.6 ± 18.3
Resource utilization band,^e^ n (%)							
Mean	3.2 ± 1.2	3.2 ± 1.1	3.1 ± 1.2	3.2 ± 1.1	<.0001	3.2 ± 1.1	3.2 ± 1.1
Comorbidity conditions, n (%)							
Acute MI	927 (5.7)	1529 (4.4)	160 (4.1)	2616 (4.7)	<.0001	73 (6.3)	2689 (4.7)
Asthma	3124 (18.2)	7794 (22.2)	861 (22.2)	11 779 (21.0)	<.0001	168 (14.6)	11 947 (20.9)
Congestive heart failure	1602 (9.5)	3022 (8.6)	301 (7.8)	4925 (8.8)	.0004	100 (8.7)	5025 (8.8)
Dementia	604 (3.6)	1325 (3.8)	177 (4.6)	2106 (3.8)	.012	42 (3.7)	2148 (3.8)
Diabetes	3598 (21.2)	7450 (21.2)	821 (21.2)	11 869 (21.2)	1.00	267 (23.2)	12 136 (21.2)
Hypertension	8594 (50.7)	17 511 (49.8)	1711 (44.1)	27 816 (49.7)	<.0001	592 (51.4)	28 408 (49.7)
Comorbidity: John Hopkin’s ADG Score^f^							
0-5	8226 (48.5)	15 966 (45.4)	1 935 (49.9)	26 127 (46.7)	<.0001	566 (49.2)	26 693 (46.7)
6-10	6279 (37.0)	13 635 (38.8)	1 368 (35.3)	21 282 (38.0)		446 (38.7)	21 728 (38.0)
>10	2455 (14.5)	5556 (15.8)	574 (14.8)	8585 (15.3)		139 (12.1)	8.724 (15.3)
Mean	6.1 ± 3.9	6.4 ± 3.9	6.0 ± 4.1	6.3 ± 3.9		6.0 ± 3.6	6.3 ± 3.9

a *P*-values were determined through ANOVA tests (continuous variables) and Chi-square tests (categorical variables).

b Additional analysis includes the other enrolment cohort that was excluded from the regression analysis as well as the total combined cohorts prior to the regression analysis (n = 57 145).

c The ON-Marg is an area-based index that illustrates the marginalization in order to better understand the discrepancies in equality across the province. The index is derived from 18 socioeconomic and demographic measures and categorized into 4 dimensions: material deprivation, dependency, residential instability, and ethnic concentration.

d Derived from the rurality index of Ontario with 0 to 9 indicating major urban centers, 10-39 indicating non-major urban centers and a score ≥40 indicating rural areas.

e Resource Utilization Bands (RUBs) is a simplified ranking system that takes into account of overall morbidity and groups them into expected use of healthcare resources. There is a total of 6 RUB groups from RUB 0 representing no resource use and an RUB of 5 and greater representing high levels of resource use.

f The Aggregated Diagnosis Groups (ADG) score is based on 5 clinical dimensions: duration of condition, severity of condition, etiology of condition, diagnostic certainty, and specialty involvement. The purpose of an ADG value is to provide a relative measure of the individual’s expected or actual health service utilization.

**Table 3.** Continuity of Care Measures.

Continuity of care measurement	Cohorts			Total combined cohort included in regression analysis N = 53 713^a^	*P*-value^b^	Additional analysis^c^	
	Team-based n = 16 247	Traditional n = 33 806	No enrolment n = 3660			Other enrolment n = 1101	Total combined cohorts N = 54 814
	N (%) *M* ± SD	N (%) *M* ± SD	N (%) *M* ± SD	N (%) *M* ± SD		N (%) *M* ± SD	N (%) *M* ± SD
Usual Provider Care (UPC)							
Mean UPC	0.54 ± 0.22	0.56 ± 0.23	0.51 ± 0.23	0.55 ± 0.22	<.0001	0.53 ± 0.22	0.55 ± 0.22
Sequential Continuity of Care (SECON)							
Mean SECON	0.50 ± 0.21	0.53 ± 0.22	0.51 ± 0.23	0.52 ± 0.22	<.0001	0.49 ± 0.22	0.52 ± 0.22
Bice-Boxerman’s Continuity of Care Index (COCI)							
Mean COC	0.36 ± 0.23	0.39 ± 0.25	0.34 ± 0.24	0.38 ± 0.24	<.0001	0.35 ± 0.23	0.38 ± 0.24

a Missing values (n = 2281; 4%) is reflected by the definitional criteria of continuity of care of at least 5 primary care visits over the past 5 years.

b *P*-values were determined through ANOVA tests (continuous variables) and Chi-square tests (categorical variables).

c Additional analysis includes the other enrolment cohort that was excluded from regression analysis as well as the total combined cohorts prior to regression analysis (n = 57 145).

**Table 4.** Family Physician (FP) and Specialist/Respirologist (SP) Visits.

Visit type	Cohorts			Total combined cohort N = 53 713^a^	*P*-value^b^	Additional analysis^c^	
	Team-based n = 16 247	Traditional n = 33 806	No enrolment n = 3660			Other enrolment n = 1101	Total combined cohorts N = 54 814
	N (%) *M* ± SD	N (%) *M* ± SD	N (%) *M* ± SD	N (%) *M* ± SD		N (%) *M* ± SD	N (%) *M* ± SD
# of FP visits	628 376 (81.3)	1 475 639 (82.5)	185 014 (82.5)	2.289 029 (82.4)	<.0001	38 356 (1.6)	2 327 385 (82.5)
# of SP visits	141 177 (18.3)	311 820 (17.5)	36 689 (16.5)	489 686 (17.6)	<.0001	4771 (1.0)	494 457 (17.5)
All visits (FP and SP) over the 5-year follow-up period							
All visits	769 553 47.4 ± 47.9	1 787 459 52.9 ± 52.6	221 703 60.6 ± 74.6	2 778 715 50.8 ± 52.6	<.0001	43 127 (1.5) 39.2 ± 42.9	2 821 842 51.5 ± 53.0
COPD-related visits^d^	34 615 (4.5) 2.1 ± 9.4	68 426 (3.8) 2.0 ± 7.9	8130 (3.7) 2.2 ± 9.1	111 171 (4.0) 2.0 ± 8.3	.22	1477 (1.3) 1.3 ± 3.9	112 648 (4.0) 2.1 ± 8.4
Mean proportions (total visits of interest/all visits over the 5 years)							
FP visits/all visits	0.84 ± 0.18	0.85 ± 0.19	0.84 ± 0.19	0.84 ± 0.19	<.0001	0.90 ± 0.12	0.84 ± 0.19
FP visits							
Mean FP Visits^e^	38.7 ± 40.1	43.7 ± 42.6	50.6 ± 64.8	42.6 ± 43.4	<.0001	34.8 ± 39.3	42.5 ± 43.8
0-17	4616 (28.4)	7564 (22.4)	929 (25.4)	13 109 (24.4)	<.0001	367 (33.3)	13 476 (24.7)
18-30	4441 (27.3)	8537 (25.2)	823 (22.5)	13 801 (25.7)	<.0001	317 (28.8)	14 118 (25.8)
31-51	3739 (23.0)	8610 (25.5)	828 (22.6)	13 177 (24.5)	<.0001	224 (20.4)	13 401 (24.5)
>51	3451 (21.2)	9095 (26.9)	1 080 (29.5)	13 626 (25.4)	<.0001	193 (17.5)	13 626 (24.9)
SP visits							
Mean SP visits^c^	8.7 ± 17.6	9.2 ± 22.4	10.0 ± 21.4	9.1 ± 20.6	.0007	4.3 ± 10.2	9.0 ± 20.8
0	3777 (23.2)	8517 (25.2)	949 (25.9)	13 243 (24.7)	<.0001	321 (29.1)	13 564 (25.1)
1-3	4668 (28.7)	9261 (27.4)	971 (26.5)	14 900 (27.7)	<.0001	430 (39.1)	15 330 (28.4)
4-10	3937 (24.2)	7722 (22.8)	788 (21.5)	12 447 (23.2)	<.0001	243 (22.1)	12 690 (23.5)
>10	3865 (23.8)	8306 (24.6)	952 (26.0)	13 123 (24.4)	<.0001	107 (9.7)	12 373 (22.9)

a Missing values (n = 2281; 4%) is reflected by the definitional criteria of having at least 5 primary care visits over the past 5 years.

b *P*-values were determined through ANOVA tests (continuous variables) and Chi-square tests (categorical variables).

c Additional analysis includes the other enrolment cohort that was excluded from regression analysis as well as the total combined cohorts prior to regression analysis (n = 57 145).

d Both non-COPD related, and COPD-related visits include PCP and SP visits.

e Represents the mean number of visits per patient over the 5-year follow-up period.

**Table 5.** Indicators of Unplanned Health Care Utilization.

Indicators of unplanned health care utilization	Cohorts			Total combined cohort N = 55 994	*P*-value^a^
	Team-based n = 16 960	Traditional n = 35 157	No enrolment n = 3877		
	N (%) *M* ± SD	N (%) *M* ± SD	N (%) *M* ± SD	N (%) *M* ± SD	
All-cause					
# of ED visits without a hospitalization	61 710 (34.4)	102 866 (57.3)	15 036 (8.4)	179 612	
Mean # of visits per patient	3.6 ± 6.1	2.9 ± 7.6	3.9 ± 12.0	3.2 ± 7.6	<.0001
Any visit	12 691 (74.8)	24 140 (68.7)	2746 (70.8)	39 577 (70.7)	<.0001
No visit	4269 (25.2)	11 017 (31.3)	1131 (29.2)	16 417 (29.3)	<.0001
# of ED visits with a hospital admission	14 968 (32.6)	27 235 (59.2)	3755 (8.2)	45 958	
Mean # of visits per patient	0.88 ± 1.8	0.77 ± 1.8	0.97 ± 2.5	0.82 ± 1.8	<.0001
Any visit	6299 (37.1)	11 506 (32.7)	1360 (35.1)	19 165 (34.2)	<.0001
No visit	10 661 (62.9)	23 651 (67.2)	2517 (64.9)	36 829 (65.8)	<.0001
# of direct hospitalizations	5298 (34.7)	8975 (58.8)	994 (6.5)	15 267	
Mean # of visits per patient	0.31 ± 0.70	0.26 ± 0.64	0.26 ± 0.63	0.27 ± 0.66	<.0001
Any visit	3777 (22.3)	6510 (18.5)	730 (18.8)	11 017 (19.7)	<.0001
No visit	13 183 (77.7)	28 647 (18.5)	3147 (81.2)	44 977 (80.3)	<.0001
# of 30-day readmissions	2943 (32.4)	5281 (58.2)	857 (9.4)	9081	
Mean # of visits per patient	0.17 ± 0.80	0.15 ± 0.72	0.22 ± 1.7	0.16 ± 0.85	<.0001
Any visit	1638 (9.7)	2949 (8.4)	370 (9.5)	4957 (8.9)	<.0001
No visit	15 322 (90.3)	32 208 (91.6)	3507 (90.5)	51 037 (91.1)	<.0001
COPD-related					
# of ED visits without a hospitalization	2994 (38.1)	4058 (51.6)	805 (10.2)	7857	
Mean # of visits per patient	0.18 ± 0.77	0.11 ± 0.71	0.21 ± 3.8	0.14 ± 1.2	<.0001
Any visit	1704 (10.1)	2298 (6.5)	301 (7.8)	4303 (7.7)	<.0001
No visit	15 256 (89.9)	32 859 (93.5)	3576 (92.2)	51 691 (92.3)	<.0001
# of ED visits with a hospital admission	1854 (36.4)	2789 (54.8)	447 (8.8)	5090	
Mean # of visits per patient	0.11 ± 0.67	0.08 ± 0.51	0.12 ± 1.0	0.09 ± 0.61	<.0001
Any visit	1088 (6.4)	1669 (4.7)	235 (6.1)	2992 (5.3)	<.0001
No visit	15 872 (93.6)	33 488 (95.3)	3642 (93.9)	53 002 (94.7)	<.0001
# of direct hospitalizations	462 (36.6)	729 (57.8)	70 (5.6)	1 261	
Mean # of visits per patient	0.03 ± 0.18	0.02 ± 0.16	0.02 ± 0.14	0.02 ± 0.16	<.0001
Any visit	423 (2.5)	664 (1.9)	66 (1.7)	1153 (2.1)	<.0001
No visit	16 537 (97.5)	34 493 (98.9)	3811 (98.3)	54 841 (97.9)	<.0001
# of 30-day readmissions	620 (33.4)	1023 (55.0)	214 (11.5)	1857	
Mean # of visits per patient	0.04 ± 0.41	0.03 ± 0.29	0.06 ± 1.4	0.03 ± 0.48	.003
Any visit	389 (2.3)	672 (1.9)	77 (2.0)	1138 (2.0)	.01
No visit	16 571 (97.7)	34 485 (98.1)	3 800 (98.0)	54 856 (98.0)	.01

a *P*-values were determined through ANOVA tests (continuous variables) and Chi-square tests (categorical variables).

**Table 6.** Relative Risk Ratios for All-cause and COPD-related Outcomes.

Cohort	Adjusted^a^ relative risk for all-cause outcomes	Adjusted relative risk for COPD-related outcomes
	RR (95% CI) *P*-value	OR (95% CI) *P*-value
ED visits without hospitalization		
Team-based^b^	Ref	Ref
Traditional^c^	1.00 (0.99-1.01) *P* = .59	1.08 (1.01-1.16) *P* = .02
No enrolment^d^	1.14 (1.12-1.17) *P* < .0001	1.75 (1.59-1.93) *P* < .0001
ED visits with hospitalization		
Team-based	Ref	Ref
Traditional	1.00 (0.97-1.02) *P* = .67	0.97 (0.89-1.06) *P* = .51
No enrolment	1.11 (1.07-1.15) *P* < .0001	1.17 (1.01-1.34) *P* = .03
Direct hospitalization		
Team-based	Ref	Ref
Traditional	0.99 (0.95-1.02) *P* = .47	1.16 (0.88-1.50) *P* = .29
No enrolment	0.94 (0.88-1.01) *P* = .08	0.53 (0.35-0.81) *P* = .003
30-day readmissions		
Team-based	Ref	Ref
Traditional	0.95 (0.90-0.99) *P* = .03	0.91 (0.79-1.06) *P* = .24
No enrolment	1.17 (1.08-1.27) *P* < .0001	2.26 (1.86-2.75) *P* < .0001

Due to the unique characteristics of the other enrolment group, it was not included in this analysis.

a Adjusted for the following covariates: sex, age, immigration status (Y/N), asthma diagnosis, ADG total, rurality, resource utilization band, marginalization summary score, continuity of care index.

b The team-based cohort included the Family Health Team.

c The traditional cohort included capitation models (ie, Family Health Networks and Family Health Organizations), Comprehensive Care Teams, and Family Health Groups.

d That is complete FFS such as walk-in clinics, unattached patients.

There is emerging, but inconsistent findings in the literature examining health care utilization within the context of Ontario primary care models.^22-25^ To our knowledge, we were not able to find publications that evaluated the impact of Ontario primary care models on unplanned health care utilization within the context of people newly diagnosed with COPD.

We tested the hypothesis that access to supports within primary care provided by team-based models could potentially lead to better disease management as demonstrated by fewer ED visits and hospitalizations.

## Methods

A retrospective cohort study was conducted using Ontario health administrative data housed within ICES. In 2018, the institute formally known as the Institute for Clinical Evaluative Sciences adopted in initialism ICES as its official name. This change acknowledges the growth and evolution of the organization’s research since its inception in 1992, while retaining the familiarity of the former acronym within the scientific community and beyond. Ontario is Canada’s largest province, with a population of 14.1 million^2^ in 2017. Ontario residents are entitled to universal public health insurance that allows for medically necessary services. These services provided under the Ontario Health Insurance Plan (OHIP) are documented in the health administrative data housed at ICES. The datasets used in this project were linked using uncoded identifiers and analysed within the ICES secure environment. ICES is an independent, non-profit research organization primarily funded by an annual grant from the Ontario Ministry of Health (MOH) and the Ministry of Long-Term Care (MLTC). As a prescribed entity under Ontario’s privacy legislation, ICES is authorized to collect and use health care data for the purpose of health system analysis, evaluation and decision support. Secure access to these data is governed by policies and procedures that are approved by the Information and Privacy Commissioner of Ontario. This project was reviewed and approved by Queen’s University Health Sciences Research Ethics Board.

**Figure 1.**
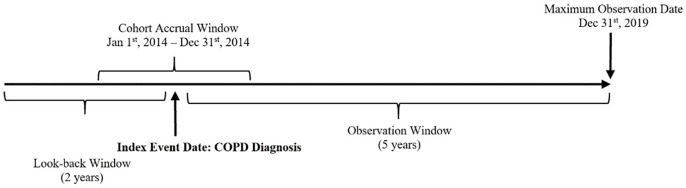
Time frame of ICES-based study.

**Figure 2.**
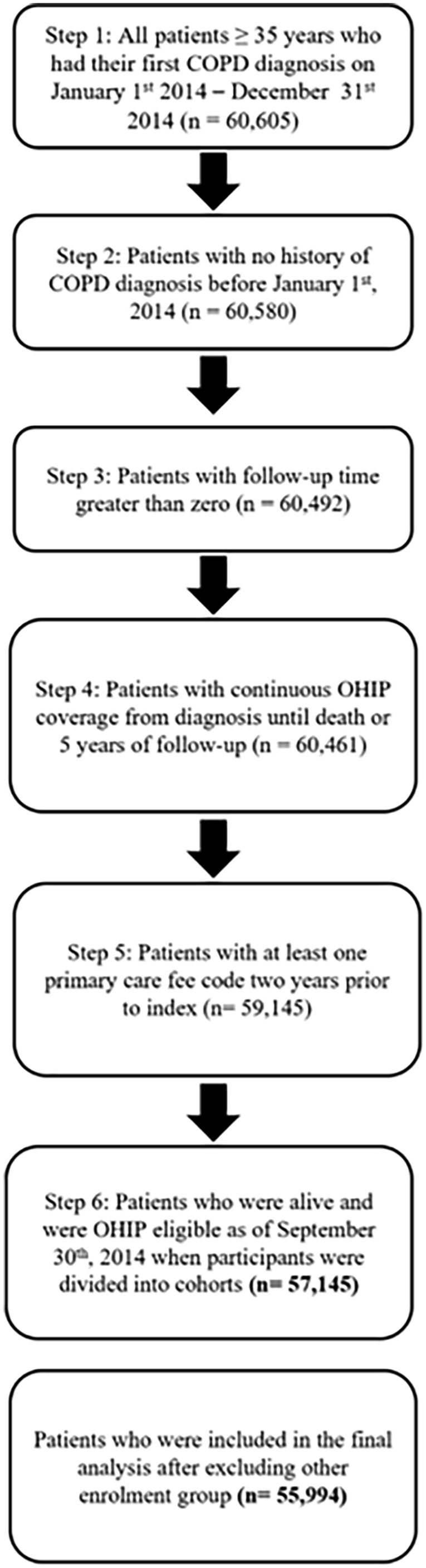
Participant flow diagram.

The study cohort included all Ontario residents with physician-diagnosed COPD from January 1st, 2014 to December 31st, 2014. The COPD diagnostic algorithm was based on a previously validated case definition derived from health administrative data. The validated case definition consists of individuals of at least 35 years and older with having 1 COPD hospitalization and/or 1 COPD ambulatory care claim using International Classification of Diseases (10th revision): J41, J43, or J44 or OHIP claims (diagnostic code: 491, 492, or 496). This case definition has a reported sensitivity of 85.0% and a specificity of 78.4%.^27,28^ As well, all participants required at least 1 primary care fee code within the past 2-year lookback period, which served as an inclusion criterion. This inclusion criterion was in place to ensure that participants had a recent interaction with primary care services. Moreover, categorization of participants into care models were determined by their primary care feed code and its corresponding FP. Individuals were followed for a maximum of 5 years from the index date, the date associated with the diagnosis of COPD (see Figure 1). The observation period was terminated for the following reasons: 1) completion of the 5-year period after the index date, or 2) upon death. The maximum follow-up date was December 31st, 2019. All individuals within the ICES database who met the inclusion and exclusion criteria were included in the cohort (n = 57 145). The participant flow diagram is illustrated in Figure 2.

Patients were assigned to primary care models based on their FP’s assignment within the Primary Care Population (PCPOP) database. Physicians in models that rostered patients were identified through the Client Agency Program Enrolment (CAPE) database. The CAPE database contains information on a patient’s association to a specific physician and primary care model. If a patient was not rostered, the patient was virtually rostered to the physician with the highest cost associated using core primary care billings. These physicians were in no enrolment models, such as walk-in clinics or solo physicians with practices completely funded through FFS. Patients were categorized at the time of the index date based on the first FP visit and its associated primary care model on September 30th, 2014. After taking into consideration the hypothesis and the distribution of cohorts, the other enrolment group, which was a unique group (n *=* 1151) that included several different primary care models (eg, Community Health Centres [CHCs], Rural and Northern Physician Agreement [RNPGA], other smaller primary care models), was not included in the regression analysis (n = 55 994). Since the purpose was to evaluate the impact of models with team-based care, the patients were then further grouped into team-based, traditional, and no enrolment.

The defined cohorts were as follows:

- **Team-based:**
  - Family Health Team (FHT)
- **Traditional (non-team based)**, which included the following remuneration models:
  - Capitation (ie, Family Health Network [FHN], Family Health Organization [FHO])
  - Comprehensive Care Model (CCM)
  - Family Health Group (FHG)
- **No enrolment (non-team based and no rostering):**
  - Physician not in an enrolment model (ie, providing care in walk-in clinics, other practices funded completely through FFS)

Given that the aim was to explore the impact of primary care models with team-based care, the team-based cohort was the reference group.

### Study Variables

#### Outcome measures

Data on hospitalizations and ED visits were taken from the Canadian Institute for Health Information—Hospital Discharge Abstract Database (CIHI-DAD) and the National Ambulatory Care Reports System (NACRS). Primary outcomes were indicators of unplanned health care utilization as defined by the following: 1) ED visits with or without subsequent hospitalization [eg, COPD-related (eg, obstructive lung disease, pneumonia), or all-cause], and 2) direct hospitalization to an acute care facility (excluding hospitalization to a psychiatric hospital). A secondary outcome included 30-day readmission after hospital discharge. Lastly, primary care visits (ie, COPD-related and all-cause) were also quantified and reported in this study. Physician specialty, specifically respirologists, was derived using the Corporate Provider Database (CPDB).

#### Covariates

There are 3 different continuity of care measures: 1) Usual Provider of Care (UPC), 2) Bice’s Continuity of Care Index (COCI), and 3) Sequential Continuity of Care (SECON). Whereas the UPC reflects the “density” of care and SECON measures the handoffs between providers, we decided to use the COCI in our regression analysis as it best reflects our intended measurement.^17,29^ The COCI considers the frequency of visits with each primary care provider along with the dispersion of visits among providers to compute a value from zero to one, where a patient receiving all primary care services through 1 provider would be assigned a value of one.^17,29^ Socio-demographic data was collected through the Ontario Registered persons Database (RPDB), Ontario Marginalization Index (ON-Marg), and Statistics Canada Census Estimates. ICD codes (versions -9, -9-CM or -10) from the Discharge Abstract Database (DAD), National Ambulatory Care Reporting System (NACRS), Same Day Surgery Database (SDS) and OHIP two years prior to the index date were used to calculate the John Hopkins Aggregated Diagnosis Group (ADG) version 11.0. The COCI was determined using diagnostic data from CIHI-DAD, OHIP, and CPDB. The adjusted models included several covariates, namely age, sex, income quintile, immigration status, rurality index, asthma diagnosis, ADG, ON-Marge score, and COCI.

### Statistical Analysis

Data analysis was conducted using the Statistical Analysis System (version 7.1). Descriptive statistics were used to summarize and describe the patient characteristics and primary care characteristics in the study. Missing outcome values were substituted with zeros prior to analysis. To assess the potential relationship between primary care practice models and unplanned health care utilization, a zero-inflated negative binomial ZINB model was applied. This model accommodates for the overdispersion of zeros, as indicated by significant Lagrange multiplier values in this study. Additionally, there were significant differences in the mean and the variance of the outcomes, which further justified the use of the ZINB model.

## Results

There were 57 145 individuals with a new physician-diagnosed COPD living in Ontario who had also received at least 1 primary care visit. After the exclusion of the other enrolment group (n = 1151), which included unique primary care models such as CHCs and RNPGA, 55 994 patients were included. Of this, 52.0% were men, 41.4% were 65 years or older, and 65.5% were from major urban areas. Most were non-immigrants (88.9%) with almost half (47.1%) of the population in the 2 lowest income quintiles (Q1 and Q2). Additionally, the ON-Marg score, which serves as a proxy measurement for socioeconomic status, averaged at 3.1 (Standard Deviation [SD] = 0.80; *P* < .0001). Almost half (49.7%) of the sample had hypertension, followed by diabetes (21.2%) and asthma (21.0%). Lastly, the resource utilization band, a simplified ranking system of overall morbidity level, was relatively similar across all cohorts (Mean [*M*] = 3.2; SD = 1.1; *P* < .0001).

All primary care visits were further differentiated into COPD-related, non-COPD related, and all-cause. Most primary care visits (96.0%) were non-COPD related. On average, the sample had 42.5 (SD = 43.8) FP visits and 9.0 (SD = 20.8) respirologist visits within the defined 5-year period. Most of the visits were with FPs, as demonstrated by a higher mean proportion of FP visits (84%) in comparison to respirologist visits (16%). Three validated measures of continuity of care were measured. All cohorts had similar average continuity of care measures.

Between 2014 and 2019, most unplanned health care utilization were ED visits without hospital admissions. There was a total of 179 612 all-cause ED visits without hospitalization (*M* = 3.2; SD = 7.6; *P* < .0001) and 45,958 all-cause ED visits with hospitalization (*M* = 0.82; SD = 1.8; *P* < .0001). There were fewer COPD-related ED visits, with 7857 COPD-related ED visits without hospitalization (*M* = 0.14; SD = 1.2; *P* < .0001) and 5090 COPD-related ED visits with hospitalization (*M* = 0.09; SD = 0.61; *P* < .0001). From the sample, 39 577 (70.7%) had an ED visit without hospitalization, 19 165 (34.2%) had an ED visit with hospitalization, 11 017 (19.7%) had a direct hospitalization, and 4957 (8.9%) had a 30-day readmission following hospital discharge during the 5-year period. Additionally, 4303 (7.7%) had a COPD-related ED visit with no hospitalization, 2992 (5.3%) had a COPD-related ED visit with hospitalization, 1153 (2.1%) had a COPD-related direct hospitalization, and 1138 (2.0%) had a COPD-related 30-day readmission.

A total of 55 994 individuals were included in the ZINB models. As mentioned previously, primary care models were categorized into 3 groups (ie, traditional, team-based, no enrolment) after careful consideration of the hypothesis. A sensitivity analysis was completed that included ZINB analyses with the other enrolment group, which provided similar results (see Supplemental Data). After adjusting for covariates, patients with newly diagnosed COPD demonstrated similar risks of experiencing all-caused unplanned health care utilization outcomes, regardless of whether they were in the team-based or traditional groups. However, the no enrolment group demonstrated a significantly higher risk of all-cause outcomes, including ED visit without hospitalization (Adjusted Relative Risks [ARR] = 1.14; CI = 1.12-1.17; *P* < .0001), ED visit with hospitalization (ARR = 1.11; CI = 1.07-1.15; *P* < .0001), and 30-day readmissions ARR = 1.17; CI = 1.08-1.27; *P* < .0001). In terms of COPD-related outcomes, the no enrolment group experienced a statistically higher risk of ED visit without hospitalization (ARR = 1.75; CI = 1.59-1.93; *P* < .0001), ED visit with hospitalization (ARR = 1.17; CI = 1.01-1.34; *P* = .03), and 30-day readmission (ARR = 2.26; CI = 1.86-2.75; *P* < .0001). The no enrolment group had a statistically reduced risk of COPD-related direct hospitalization (ARR = 0.53; CI = 0.35-0.81; *P* = .003).

## Discussion

We conducted a population-based retrospective cohort study using Ontario health administrative data and found that there was minimal difference in the adjusted relative risk of experiencing an all-cause outcome or COPD-related outcomes for patients in either the team-based or traditional group. As expected, the no enrolment group, which represented largely the unattached population in Ontario, had an increased risk of unplanned health care utilization (ie, ED visit with or without hospitalizations, 30-day readmissions) for most outcomes, with the exception of COPD-related direct hospitalizations. This finding is consistent given that the unattached patients often rely on alternative services, such as the ED, as a substitute for primary care. Consequently, unattached patients are at an increased risk of a COPD-related ED visit with hospitalization, while their risk of experiencing a COPD-related direct hospitalization is reduced. Overall, unplanned health care utilization was high, with 70.7% of the sample having at least 1 ED visit without a subsequent hospitalization within a 5-year period, and 34.2% having at least 1 ED visit resulting in a hospitalization.

The findings of this study are partially consistent with previous research examining health service utilization use among different primary care models in Ontario.^22-25^ However, comparing our findings to existing literature was challenging due to the variations in the reference group used and the focus on the general population in previous studies. Prior to the implementation of team-based models (eg, FHT) in 2006, 1 study showed that patients in the capitation model had higher rates of ED visits when compared to enhanced FFS (Adjusted Relative Risk [ARR] = 1.20; 95% CI = 1.15-1.25).^22^ Studies completed after the introduction of team-based models reported mixed results. Glazier et al^23^ reported slightly higher overall ED use in the team-based model compared to FFS and FHOs between 2004/05 to 2011/2012. However, the longitudinal analysis demonstrated that all models had similar levels of unplanned health care utilization. In contrast, our findings diverged from research studies investigating unplanned health care use in the context of team-based models in other Canadian provinces.^30-33^ However, differences in methodology likely contributed to these contrasting results. A systematic review (*n* = 14) encompassing 3 studies investigating the impact of team-based primary care models on health care utilization found that such models led to a reduction in ED visits in Quebec and Alberta.^30-33^ First, it is important to note that the studies were conducted in other Canadian provinces, which is distinct from our study completed in Ontario. Therefore, unique features such as funding and organizational structures in primary health care systems may have contributed to the varying outcomes. Second, studies by Héroux et al^30^ and Campbell et al^31^ focused solely on vulnerable population, which could have significantly influenced the decrease in ED visits. Third, studies by Campbell et al^31^ and Manns et al^32^ were completed in the context of patients with diabetes only. Patients with diabetes require fundamentally different care compared to patients with COPD, making it challenging to generalize these findings.

There were some demographic differences across groups with statistical significance; however, it is unlikely that they were clinically meaningful. Specifically, the team-based group had a higher proportion of patients residing in non-major urban (37.5%) and rural (20.1%) areas (*P* < .0001). Moreover, the mean rurality index was double (*M* = 21.2; SD = 22.5) in the team-based group when compared to the traditional group (*M* = 9.1; SD = 13.4) and the no enrolment group (*M* = 10.4; SD = 18.2) with a *P* value <.0001. Within the analysis, non-major urban (Rurality Index of Ontario [RIO] = 10-39) and rural areas (RIO > 39) are significant factors that increased the risk of unplanned health care utilization. Patients in rural areas often rely more on EDs to receive essential care due to limited access to primary health care.^34^ Additionally, patients in rural areas are at a higher risk of ACSC hospitalization.^25^ The team-based group also had a higher proportion of patients who were non-immigrants (96.5%) compared to the traditional group (85.3%) and no enrolment group (88.3%) (*P* < .0001). One concern with team-based models is their potential bias towards serving higher-incoming neighborhoods, fewer proportion of immigrants in the roster, overall lower use of the health care system and lower comorbidity despite often located in rural areas.^24^ Furthermore, the relationship between immigration and health care utilization is complex, with implications from varying socioeconomic status and barriers to accessing health care.^35^ The different proportions of immigrants among cohorts were controlled for in the analysis. However, these population differences should be further examined within the context of strategies to promote equitable care for all.

It could also be argued that higher COPD-related hospitalizations observed in the team-based cohort could be a consequence of better self-management, fostered through team-based care. However, it should be noted that administrative databases cannot determine the “appropriateness” of unplanned health care utilization or differentiate whether such visit could have been prevented through self-management interventions. Nevertheless, a systematic review (*n* [number of studies] = 184) on self-management interventions, including patient education, support for decision making, and self-monitoring, demonstrated a small reduction in unplanned health care utilization.^36^ In contrast, another systematic review and meta-analysis (*n* [number of studies] = 49) on international chronic disease self-management programs showed mixed results regarding its effect on general health care utilization.^37^ A report by Glazier et al^24^ found that between 2008/09 to 2009/10, patients receiving primary care from “other” models, FHGs, and CHCs were associated with lower ED visits, while team-based practice models (ie, FHTs) were associated with higher ED visits after adjustments. Similarly, in a 1-year study, patients in blended-capitation models with team-based care had a higher risk of an ambulatory care sensitive conditions hospitalization (AOR = 1.06; 95% CI = 1.00-1.12).^25^ The team-based group in the 1-year study also had a higher rurality index score, indicating potential access implications that warrant further analysis. The relationship between self-management and health care utilization remains ambiguous, and it cannot be definitively concluded that higher ED visits were a directly caused by improved self-management.

The results may also be influenced by various physician remuneration methods, which was not further explored in this study. For example, traditional models may receive bonuses and incentives that focus on providing preventative care and managing chronic diseases.^15,19^ These differences in remuneration incentives could have implications for patient outcomes and should be considered within the context of this study. Another example is the use of capitation models, which incentivizes referrals to other care providers and may in turn improve chronic disease management.^21^ However, in this study, this was indirectly measured through respirologist visits and the SECON score, which assessed handoffs between FPs and respirologists. Minimal differences were found in specialist visits and the SECON value among the cohorts. It is possible that the patients included in this study sought care from other specialists, which was not accounted for and may have contributed to the findings.

Our study analysis accounted for several patient characteristics. However, most of the covariates did not exhibit a statistically significant association with unplanned health care utilization in the ZINB models and the observed pattern of association were inconsistent and inconclusive. Notably, patients with low COCI scores were more likely to experience adverse outcomes and this association was frequently statistically significant. The inclusion of the COCI scores, given the complexity of COPD management, raises the question as to whether the provision of continuous care may be a characteristic of a team-based care, potentially acting as a confounder and part of the causal pathway. To address this concern, a sensitivity analysis excluding COCI as a covariate was performed, and the adjustive relative risk ratios were compared. The differences in adjusted relative risk estimates between the 2 models were below 10%, indicating that the associations between the practice model and outcomes remained consistent regardless of the influence of COCI. Lastly, concomitant asthma and COPD is of particular concern, as the estimated prevalence of the asthma-COPD overlap is somewhere between 4 to 12%^38^ and is associated with greater comorbidity burden, medication use, and cost to the health care system.^39,40^ However, when asthma was included as a covariate, the relative risk estimates did not change, suggesting that a COPD diagnosis had an independent effect.

### Strengths and Limitations

The study has several strengths, including its population-based approach, utilization of a validated COPD definition, and a comprehensive capture of health service use in individuals with newly diagnosed COPD. The adjustment of validated covariates in all analyses further enhances the reliability of the results. However, there are limitations to this study, in part due to the use of health administrative data. First, while physician-diagnosed COPD is a validated method, there is a possibility of misclassification. Underdiagnosis of OCPD using physician-diagnosed COPD has been consistently observed, leading to underestimated prevalence; however, underdiagnosis has been a consistent finding over the years, and therefore it is unlikely to have a significant impact on the findings.^27^ Second, the exclusion of CHCs and other enrolment group (ie, RNPGAs) due to the methodology used to create cohorts limits the generalizability of the results. Most studies in the literature reviewed included CHCs in their cohort; therefore, no further comments can be made on CHCs and RNPGAs. Third, health administrative data only includes care received from physicians and does not provide information on care provided by key health care professionals such as registered nurses, nurse practitioners, or COPD educators. Allied health professionals play a crucial role in chronic disease management and their individual impact could not be captured in the analysis. However, their contribution would be indirectly captured within the team-based practice model. Future studies should explore the relationship between primary care models and indicators of unplanned health care utilization in the context of potential influencing factors (eg, nurse practitioner involvement, incentives for chronic disease management) that were not accounted for in this study.

It is important to acknowledge the imbalance in the number of patients across the groups, with the majority (62.8%) in the traditional group, which represented 3 primary care models (capitation, FHG, CCM). Larger sample sizes are often preferred for their validity and better representation. This disparity may be attributed to physician preferences for certain models based on difference incentives (ie, improved quality of work life balance). Although there are minimal differences among cohorts in terms of patient and primary care characteristics, the results should be interpreted within the context of cohort sizes. COPD severity on the index date could not be included due to limitations in methodology. Although age was controlled for in the analysis, serving as an indirect measurement for COPD severity, it is important to recognize that our study can not provide insights related to the potential impact of COPD severity on observed outcomes. Finally, while COPD is considered an ambulatory care sensitive condition, it is important to recognize that a proportion of ED visits and hospitalizations may not be avoidable. The methodology used does not allow for differentiation of a “truly avoidable” unplanned health care utilization, and therefore the distribution of such avoidable health care utilization across the cohorts remain largely unknown.

## Conclusion

Primary care reforms and their impact on quality and accessibility of primary care services is an important discussion. Studies that included a comparison of outcomes across Ontario primary care models have reported inconsistent results.^22-25^ The complexity of primary care models, influenced by remuneration packages and organizational structures, underscores the need for ongoing research to enhance our overall understanding of primary care reforms. This study offers a unique perspective by focusing on the impact of team-based primary care models on patients with newly diagnosed COPD, providing valuable insights into the care received during the early years following diagnosis. In summary, despite adjusting for covariates, this study suggests that the current organization and operationalization of team-based models do not reduce the risk of unplanned health care utilization compared to traditional models. Most importantly, our study supports the notion that limited access to primary care, such as through episodic care or walk-ins only, increases the risk of all-cause unplanned health care utilization and ultimately increase total spending costs.

The study has identified several research gaps that warrant attention. As we continue to pursue primary health care reforms, it is imperative to monitor, evaluate, and conduct further research on the impact of these reforms. Conceptually and logically, primary care team-based models are well positioned to provide comprehensive and accessible care. Our findings suggest that teams may not be optimized to provide the level of comprehensive and integrated care. It is important to note that our study does not imply that team-based primary care models inherently lead to less unplanned health care utilization, rather it highlights the potential for improvement within the existing primary care model frameworks.

## Supplemental Material

sj-docx-1-jpc-10.1177_21501319231201080 – Supplemental material for The Impact of Primary Care Practice Models on Indicators of Unplanned Health Care Utilization for Ontario Adults Newly Diagnosed With Chronic Obstructive Pulmonary Disease: A Retrospective Cohort Study Click here for additional data file. Supplemental material, sj-docx-1-jpc-10.1177_21501319231201080 for The Impact of Primary Care Practice Models on Indicators of Unplanned Health Care Utilization for Ontario Adults Newly Diagnosed With Chronic Obstructive Pulmonary Disease: A Retrospective Cohort Study by Ruixi Sheng, Joan E Tranmer, Christina Godfrey and Thomas Rotter in Journal of Primary Care & Community Health

sj-docx-2-jpc-10.1177_21501319231201080 – Supplemental material for The Impact of Primary Care Practice Models on Indicators of Unplanned Health Care Utilization for Ontario Adults Newly Diagnosed With Chronic Obstructive Pulmonary Disease: A Retrospective Cohort Study Click here for additional data file. Supplemental material, sj-docx-2-jpc-10.1177_21501319231201080 for The Impact of Primary Care Practice Models on Indicators of Unplanned Health Care Utilization for Ontario Adults Newly Diagnosed With Chronic Obstructive Pulmonary Disease: A Retrospective Cohort Study by Ruixi Sheng, Joan E Tranmer, Christina Godfrey and Thomas Rotter in Journal of Primary Care & Community Health

sj-docx-3-jpc-10.1177_21501319231201080 – Supplemental material for The Impact of Primary Care Practice Models on Indicators of Unplanned Health Care Utilization for Ontario Adults Newly Diagnosed With Chronic Obstructive Pulmonary Disease: A Retrospective Cohort Study Click here for additional data file. Supplemental material, sj-docx-3-jpc-10.1177_21501319231201080 for The Impact of Primary Care Practice Models on Indicators of Unplanned Health Care Utilization for Ontario Adults Newly Diagnosed With Chronic Obstructive Pulmonary Disease: A Retrospective Cohort Study by Ruixi Sheng, Joan E Tranmer, Christina Godfrey and Thomas Rotter in Journal of Primary Care & Community Health
