# Supplementary material for: The Impact of Primary Care Practice Models on Indicators of Unplanned Health Care Utilization for Ontario Adults Newly Diagnosed With Chronic Obstructive Pulmonary Disease: A Retrospective Cohort Study
Source: J Prim Care Community Health. 2023 Sep 22;14:21501319231201080. doi: 10.1177/21501319231201080 (PMC10517618; doi:10.1177/21501319231201080)
Supplement: sj-docx-1-jpc-10.1177_21501319231201080 – Supplemental material for The Impact of Primary Care Practice Models on Indicators of Unplanned Health Care Utilization for Ontario Adults Newly Diagnosed With Chronic Obstructive Pulmonary Disease: A Retrospective Cohort Study [file sj-docx-1-jpc-10.1177_21501319231201080.docx]

**Sensitivity Analysis ZINB Model with Other Group**

**Table 1**

*Summary of results from the zero-inflated negative binomial model, all-cause ED visits without hospitalization*

| Parameter | ZINB | | | Prob Chi Sq |
| --- | --- | --- | --- | --- |
|  | RR | RR Lower | RR Upper |  |
| Traditional | 1.00 | 0.99 | 1.01 | 0.813 |
| No enrolment | 1.14 | 1.12 | 1.16 | <0.0001 |
| Other Group | 0.86 | 0.83 | 0.89 | <0.0001 |
| Team based | 1 | 1 | 1 |  |
| Sex F | 0.94 | 0.93 | 0.95 | <0.0001 |
| Sex M | 1 | 1 | 1 |  |
| Patient age 50-64 | 0.87 | 0.86 | 0.88 | <0.0001 |
| Patient age 65-74 | 0.78 | 0.77 | 0.79 | <0.0001 |
| Patient age 75-84 | 0.82 | 0.81 | 0.83 | <0.0001 |
| Patient age 85+ | 0.85 | 0.83 | 0.87 | <0.0001 |
| Patient age 34-49 | 1 | 1 | 1 |  |
| ADG Total Score | 1.09 | 1.09 | 1.09 | <0.0001 |
| Asthma prevalence | 1.05 | 1.03 | 1.06 | <0.0001 |
| Income Quintile 2 | 0.90 | 0.89 | 0.91 | <0.0001 |
| Income Quintile 3 | 0.90 | 0.88 | 0.92 | <0.0001 |
| Income Quintile 4 | 0.85 | 0.84 | 0.87 | <0.0001 |
| Income Quintile 5 | 0.83 | 0.82 | 0.85 | <0.0001 |
| Income Quintile 1 | 1 | 1 | 1 |  |
| Rurality index 2008 | 1.01 | 1.01 | 1.01 | <0.0001 |
| Resource utilization band | 1.00 | 0.99 | 1.01 | 0.56 |
| ONMARG Summary Score | 1.09 | 1.08 | 1.10 | <0.0001 |
| Immigration 1 | 0.82 | 0.80 | 0.83 | <0.0001 |
| Immigration 0 | 1 | 1 | 1 |  |
| COCI missing | 0.76 | 0.72 | 0.81 | <0.0001 |
| COCI 0 – 0.20 | 2.43 | 2.39 | 2.46 | <0.0001 |
| COCI 0.21 – 0.30 | 1.63 | 1.60 | 1.66 | <0.0001 |
| COCI 0.31 – 0.50 | 1.34 | 1.31 | 1.36 | <0.0001 |
| COCI 0.51-1.0 | 1 | 1 | 1 | 1 |

Lagrange Multiplier Statistics: Chi-Square value of 3590.1; Pr>ChiSq < 0.0001

**Table 2**

*Summary of results from the zero-inflated negative binomial model, all-cause ED visits with hospitalization*

| Parameter | ZINB | | | Prob Chi Sq |
| --- | --- | --- | --- | --- |
|  | RR | RR Lower | RR Upper |  |
| Traditional | 1.00 | 0.98 | 1.02 | 0.671 |
| No enrolment | 1.11 | 1.07 | 1.15 | <0.0001 |
| Other | 0.82 | 0.77 | 0.89 | <0.0001 |
| Team based | 1 | 1 | 1 |  |
| Sex F | 0.96 | 0.94 | 0.98 | <0.0001 |
| Sex M | 1 | 1 | 1 |  |
| Patient age 50-64 | 1.09 | 1.06 | 1.13 | <0.0001 |
| Patient age 65-74 | 1.20 | 1.15 | 1.24 | <0.0001 |
| Patient age 75-84 | 1.34 | 1.29 | 1.39 | <0.0001 |
| Patient age 85+ | 1.53 | 1.47 | 1.58 | <0.0001 |
| Patient age 34-49 | 1 | 1 | 1 |  |
| ADG Total Score | 1.03 | 1.02 | 1.03 | <0.001 |
| Asthma prevalence | 0.94 | 0.92 | 0.97 | <0.001 |
| Income Quintile 2 | 0.95 | 0.92 | 0.97 | 0.0001 |
| Income Quintile 3 | 0.98 | 0.94 | 1.01 | 0.158 |
| Income Quintile 4 | 0.98 | 0.95 | 1.02 | 0.443 |
| Income Quintile 5 | 0.98 | 0.94 | 1.03 | 0.431 |
| Income Quintile 1 | 1 | 1 | 1 |  |
| Rurality index 2008 | 1.00 | 1.00 | 1.00 | <0.0001 |
| Resource utilization band | 1.08 | 1.06 | 1.09 | <0.0001 |
| ONMARG Summary Score | 1.07 | 1.05 | 1.1 | <0.0001 |
| Immigration 1 | 0.93 | 0.90 | 0.97 | 0.0003 |
| Immigration 0 | 1 | 1 | 1 |  |
| COCI missing | 1.86 | 1.50 | 2.30 | <0.0001 |
| COCI 0 – 0.20 | 2.38 | 2.29 | 2.47 | <0.0001 |
| COCI 0.21 – 0.30 | 1.53 | 1.46 | 1.59 | <0.0001 |
| COCI 0.31 – 0.50 | 1.25 | 1.20 | 1.30 | <0.0001 |
| COCI 0.51-1.0 | 1.00 | 1.00 | 1.00 |  |

Lagrange Multiplier Statistics: Chi-Square value of 1787.41; Pr>ChiSq < 0.0001

**Table 3**

*Summary of results from the zero-inflated negative binomial model, all -cause direct hospitalization*

| Parameter | ZINB | | | Prob Chi Sq |
| --- | --- | --- | --- | --- |
|  | RR | RR Lower | RR Upper |  |
| Traditional | 0.99 | 0.95 | 1.02 | 0.0.521 |
| No enrolment | 0.94 | 0.88 | 1.01 | 0.092 |
| Other | 0.93 | 0.83 | 1.05 | 0.260 |
| Team based | 1 | 1 | 1 |  |
| Sex F | 0.95 | 0.92 | 0.98 | 0.002 |
| Sex M | 1 | 1 | 1 |  |
| Patient age 50-64 | 1.07 | 1.02 | 1.13 | 0.008 |
| Patient age 65-74 | 1.09 | 1.03 | 1.15 | 0.002 |
| Patient age 75-84 | 1.10 | 1.04 | 1.17 | 0.002 |
| Patient age 85+ | 1.16 | 1.06 | 1.28 | 0.002 |
| Patient age 34-49 | 1 | 1 | 1 |  |
| ADG Total Score | 1.01 | 1.00 | 1.01 | 0.407 |
| Asthma prevalence | 0.95 | 0.92 | 0.99 | 0.022 |
| Income Quintile 2 | 1.02 | 0.97 | 1.08 | 0.378 |
| Income Quintile 3 | 1.00 | 0.94 | 1.06 | 0.934 |
| Income Quintile 4 | 0.99 | 0.92 | 1.05 | 0.677 |
| Income Quintile 5 | 0.97 | 0.90 | 1.04 | 0.378 |
| Income Quintile 1 | 1 | 1 | 1 |  |
| Rurality index 2008 | 1 | 1 | 1 | 0.601 |
| Resource utilization band | 1.04 | 1.01 | 1.06 | 0.030 |
| ONMARG Summary Score | 1.00 | 0.97 | 1.03 | 0.931 |
| Immigration 1 | 0.98 | 0.92 | 1.05 | 0.561 |
| Immigration 0 | 1 | 1 | 1 |  |
| COCI missing | 1.08 | 0.88 | 1.34 | 0.449 |
| COCI 0 – 0.20 | 1.33 | 1.27 | 1.39 | <0.0001 |
| COCI 0.21 – 0.30 | 1.13 | 1.07 | 1.19 | <0.0001 |
| COCI 0.31 – 0.50 | 1.07 | 1.02 | 1.13 | 0.006 |
| COCI 0.51-1.0 | 1 | 1 | 1 |  |

Lagrange Multiplier Statistics: Chi-Square value of 2050.18; Pr>ChiSq < 0.0001

**Table 4**

*Summary of results from the zero-inflated negative binomial model, all-cause 30-day readmissions*

| Parameter | ZINB | | | Prob Chi Sq |
| --- | --- | --- | --- | --- |
|  | RR | RR Lower | RR Upper |  |
| Traditional | 0.95 | 0.90 | 1.00 | 0.037 |
| No enrolment | 1.18 | 1.09 | 1.28 | <0.0001 |
| Other | 0.88 | 0.74 | 1.05 | 0.161 |
| Team based | 1 | 1 | 1 |  |
| Sex F | 1.00 | 0.96 | 1.04 | 0.938 |
| Sex M | 1 | 1 | 1 |  |
| Patient age 50-64 | 0.92 | 0.86 | 0.99 | 0.036 |
| Patient age 65-74 | 0.85 | 0.79 | 0.92 | <0.0001 |
| Patient age 75-84 | 0.86 | 0.79 | 0.93 | 0.0002 |
| Patient age 85+ | 0.89 | 0.81 | 0.98 | 0.016 |
| Patient age 34-49 | 1 | 1 | 1 |  |
| ADG Total Score | 1.02 | 1.01 | 1.03 | <0.001 |
| Asthma prevalence | 0.94 | 0.89 | 0.99 | 0.017 |
| Income Quintile 2 | 0.98 | 0.92 | 1.05 | 0.560 |
| Income Quintile 3 | 1.03 | 0.95 | 1.11 | 0.465 |
| Income Quintile 4 | 1.10 | 1.01 | 1.20 | 0.035 |
| Income Quintile 5 | 1.06 | 0.96 | 1.17 | 0.233 |
| Income Quintile 1 | 1 | 1 | 1 |  |
| Rurality index 2008 | 1 | 1 | 1 | 0.583 |
| Resource utilization band | 1.03 | 0.99 | 1.06 | 0.104 |
| ONMARG Summary Score | 1.11 | 1.06 | 1.16 | <0.0001 |
| Immigration 1 | 0.99 | 0.91 | 1.07 | 0.799 |
| Immigration 0 | 1 | 1 | 1 |  |
| COCI missing | 1.01 | 0.38 | 2.71 | 0.980 |
| COCI 0 – 0.20 | 1.90 | 1.72 | 2.10 | <0.0001 |
| COCI 0.21 – 0.30 | 1.35 | 1.21 | 1.51 | <0.0001 |
| COCI 0.31 – 0.50 | 1.18 | 1.05 | 1.32 | 0.0055 |
| COCI 0.51-1.0 | 1.00 | 1.00 | 1.00 |  |

Lagrange Multiplier Statistics: Chi-Square value of 235.91; Pr>ChiSq < 0.0001

**Table 5**

*Summary of results from the zero-inflated negative binomial model, COPD-related ED visits without hospitalization*

| Parameter | ZINB | | | Prob Chi Sq |
| --- | --- | --- | --- | --- |
|  | RR | RR Lower | RR Upper |  |
| Traditional | 1.08 | 1.01 | 1.16 | 0.023 |
| No enrolment | 1.75 | 1.59 | 1.93 | <0.0001 |
| Other | 0.57 | 0.44 | 0.74 | <0.0001 |
| Team based | 1 | 1 | 1 |  |
| Sex F | 0.88 | 0.83 | 0.94 | <0.0001 |
| Sex M | 1 | 1 | 1 |  |
| Patient age 50-64 | 1.19 | 1.08 | 1.30 | 0.0003 |
| Patient age 65-74 | 1.05 | 0.94 | 1.16 | 0.399 |
| Patient age 75-84 | 0.88 | 0.78 | 0.00 | 0.0273 |
| Patient age 85+ | 0.81 | 0.67 | 0.97 | 0.0251 |
| Patient age 34-49 | 1 | 1 | 1 |  |
| ADG Total Score | 1.01 | 1.00 | 1.02 | 0.190 |
| Asthma prevalence | 1.00 | 0.93 | 1.07 | 0.921 |
| Income Quintile 2 | 0.95 | 0.87 | 1.03 | 0.192 |
| Income Quintile 3 | 0.91 | 0.82 | 1.01 | 0.079 |
| Income Quintile 4 | 0.90 | 0.79 | 1.04 | 0.126 |
| Income Quintile 5 | 0.94 | 0.81 | 1.09 | 0.410 |
| Income Quintile 1 | 1 | 1 | 1 |  |
| Rurality index 2008 | 1.01 | 1.00 | 1.01 | <0.0001 |
| Resource utilization band | 1.02 | 0.98 | 1.06 | 0.349 |
| ONMARG Summary Score | 1.19 | 1.12 | 1.27 | <0.0001 |
| Immigration 1 | 0.68 | 0.56 | 0.82 | <0.0001 |
| Immigration 0 | 1 | 1 | 1 |  |
| COCI missing | 1.18 | 0.61 | 2.30 | 0.626 |
| COCI 0 – 0.20 | 3.45 | 2.95 | 4.03 | <0.0001 |
| COCI 0.21 – 0.30 | 2.08 | 1.76 | 2.46 | <0.0001 |
| COCI 0.31 – 0.50 | 1.66 | 1.39 | 1.97 | <0.0001 |
| COCI 0.51-1.0 | 1 | 1 | 1 | 1 |

Lagrange Multiplier Statistics: Chi-Square value of 247.20; Pr>ChiSq < 0.0001

**Table 6**

*Summary of results from the zero-inflated negative binomial model, COPD-related ED visits with hospitalization*

| Parameter | ZINB | | | Prob Chi Sq |
| --- | --- | --- | --- | --- |
|  | RR | RR Lower | RR Upper |  |
| Traditional | 0.98 | 0.90 | 1.07 | 0.616 |
| No enrolment | 1.17 | 1.02 | 1.35 | 0.024 |
| Other | 0.72 | 0.49 | 1.05 | 0.085 |
| Team based | 1 | 1 | 1 |  |
| Sex F | 1.01 | 0.93 | 1.09 | 0.878 |
| Sex M | 1 | 1 | 1 |  |
| Patient age 50-64 | 0.87 | 0.75 | 1.00 | 0.057 |
| Patient age 65-74 | 0.73 | 0.63 | 0.86 | <0.0001 |
| Patient age 75-84 | 0.73 | 0.63 | 0.86 | <0.0001 |
| Patient age 85+ | 0.78 | 0.63 | 0.93 | 0.0061 |
| Patient age 34-49 | 1 | 1 | 1 |  |
| ADG Total Score | 1.00 | 0.99 | 1.02 | 0.930 |
| Asthma prevalence | 0.90 | 0.82 | 0.99 | 0.029 |
| Income Quintile 2 | 0.80 | 0.71 | 0.90 | 0.0001 |
| Income Quintile 3 | 0.74 | 0.64 | 0.85 | <0.0001 |
| Income Quintile 4 | 0.79 | 0.67 | 0.94 | 0.0073 |
| Income Quintile 5 | 0.82 | 0.68 | 0.99 | 0.0342 |
| Income Quintile 1 | 1 | 1 | 1 |  |
| Rurality index 2008 | 1 | 0.99 | 1 | 0.012 |
| Resource utilization band | 0.98 | 0.93 | 1.03 | 0.432 |
| ONMARG Summary Score | 1.01 | 0.93 | 1.09 | 0.886 |
| Immigration 1 | 1.02 | 0.85 | 1.23 | 0.8036 |
| Immigration 0 | 1 | 1 | 1 |  |
| COCI missing | 0.05 | 0.01 | 0.37 | 0.0033 |
| COCI 0 – 0.20 | 4.06 | 2.99 | 5.51 | <0.0001 |
| COCI 0.21 – 0.30 | 2.02 | 1.47 | 2.79 | <0.0001 |
| COCI 0.31 – 0.50 | 1.49 | 1.07 | 2.08 | 0.020 |
| COCI 0.51-1.0 | 1 | 1 | 1 | 1 |

Lagrange Multiplier Statistics: Chi-Square value of 202.41 Pr>ChiSq < 0.0001

**Table 7**

*Summary of results from the zero-inflated negative binomial model, COPD-related direct hospitalization*

| Parameter | ZINB | | | Prob Chi Sq |
| --- | --- | --- | --- | --- |
|  | RR | RR Lower | RR Upper |  |
| Traditional | 1.30 | 1.09 | 1.56 | 0.0035 |
| No enrolment | 1.24 | 0.83 | 1.83 | 0.2944 |
| Other | 2.16 | 1.44 | 3.25 | 0.0002 |
| Team based | 1 | 1 | 1 |  |
| Sex F | 0.76 | 0.65 | 0.89 | 0.0008 |
| Sex M | 1 | 1 | 1 |  |
| Patient age 50-64 | 2.74 | 2.07 | 3.63 | < 0.0001 |
| Patient age 65-74 | 3.54 | 2.63 | 4.77 | < 0.0001 |
| Patient age 75-84 | 3.23 | 2.36 | 4.42 | < 0.0001 |
| Patient age 85+ | 3.04 | 2.18 | 4.23 | < 0.0001 |
| Patient age 34-49 | 1 | 1 | 1 |  |
| ADG Total Score | 1.02 | 0.99 | 1.05 | 0.121 |
| Asthma prevalence | 1.06 | 0.87 | 1.27 | 0.573 |
| Income Quintile 2 | 0.88 | 0.69 | 1.13 | 0.20 |
| Income Quintile 3 | 1.08 | 0.80 | 1.45 | 0.621 |
| Income Quintile 4 | 0.58 | 0.41 | 0.80 | 0.0009 |
| Income Quintile 5 | 0.73 | 0.48 | 1.11 | 0.143 |
| Income Quintile 1 | 1 | 1 | 1 |  |
| Rurality index 2008 | 1.01 | 1.01 | 1.01 | < 0.0001 |
| Resource utilization band | 0.96 | 0.86 | 1.07 | 0.4305 |
| ONMARG Summary Score | 1.02 | 0.86 | 1.20 | 0.824 |
| Immigration 1 | 0.49 | 0.36 | 0.66 | < 0.0001 |
| Immigration 0 | 1 | 1 | 1 |  |
| COCI missing | 15.75 | 5.23 | 47.45 | < 0.0001 |
| 2.46 | 1.89 | 1.89 | 3.20 | <0.0001 |
| COCI 0.21 – 0.30 | 1.56 | 1.15 | 2.10 | 0.004 |
| COCI 0.31 – 0.50 | 1.52 | 1.13 | 2.05 | 0.005 |
| COCI 0.51-1.0 | 1 | 1 | 1 | 1 |

Lagrange Multiplier Statistics: Chi-Square value of 9.668 Pr>ChiSq 0.0009

**Table 8**

*Summary of results from the zero-inflated negative binomial model, COPD-related 30-day readmission*

| Parameter | ZINB | | | Prob Chi Sq |
| --- | --- | --- | --- | --- |
|  | RR | RR Lower | RR Upper |  |
| Traditional | 0.92 | 0.79 | 1.07 | 0.266 |
| No enrolment | 2.27 | 1.87 | 2.75 | <0.0001 |
| Other | 0.62 | 0.35 | 1.08 | 0.0924 |
| Team based | 1 | 1 | 1 |  |
| Sex F | 1.21 | 1.06 | 1.38 | 0.0044 |
| Sex M | 1 | 1 | 1 |  |
| Patient age 50-64 | 0.67 | 0.53 | 0.84 | 0.0005 |
| Patient age 65-74 | 0.35 | 0.27 | 0.45 | <0.0001 |
| Patient age 75-84 | 0.40 | 0.31 | 0.51 | <0.0001 |
| Patient age 85+ | 0.42 | 0.2931 | 0.57 | <0.0001 |
| Patient age 34-49 | 1 | 1 | 1 |  |
| ADG Total Score | 0.97 | 0.95 | 0.99 | 0.020 |
| Asthma prevalence | 0.78 | 0.66 | 0.92 | 0.0027 |
| Income Quintile 2 | 0.89 | 0.74 | 1.07 | 0.222 |
| Income Quintile 3 | 0.83 | 0.65 | 1.05 | 0.126 |
| Income Quintile 4 | 1.38 | 1.00 | 1.90 | 0.047 |
| Income Quintile 5 | 1.32 | 1.00 | 1.90 | 0.0473 |
| Income Quintile 1 | 1 | 1 | 1 |  |
| Rurality index 2008 | 1 | 0.99 | 1.00 | 0.392 |
| Resource utilization band | 1.08 | 0.99 | 1.17 | 0.0746 |
| ONMARG Summary Score | 1.35 | 1.19 | 1.55 | <0.0001 |
| Immigration 1 | 0.71 | 0.50 | 1.01 | 0.0561 |
| Immigration 0 | 1 | 1 | 1 |  |
| COCI missing | 1.70 | 0.13 | 21.74 | 0.681 |
| COCI 0 – 0.20 | 2.83 | 1.58 | 5.05 | 0.131 |
| COCI 0.21 – 0.30 | 1.59 | 0.87 | 2.92 | 0.131 |
| COCI 0.31 – 0.50 | 0.97 | 0.51 | 1.85 | 0.937 |
| COCI 0.51-1.0 | 1 | 1 | 1 | 1 |

Lagrange Multiplier Statistics: Chi-Square value of 85.25 Pr>ChiSq < 0.0001
