# Supplementary material for: The Impact of Primary Care Practice Models on Indicators of Unplanned Health Care Utilization for Ontario Adults Newly Diagnosed With Chronic Obstructive Pulmonary Disease: A Retrospective Cohort Study
Source: J Prim Care Community Health. 2023 Sep 22;14:21501319231201080. doi: 10.1177/21501319231201080 (PMC10517618; doi:10.1177/21501319231201080)
Supplement: sj-docx-3-jpc-10.1177_21501319231201080 – Supplemental material for The Impact of Primary Care Practice Models on Indicators of Unplanned Health Care Utilization for Ontario Adults Newly Diagnosed With Chronic Obstructive Pulmonary Disease: A Retrospective Cohort Study [file sj-docx-3-jpc-10.1177_21501319231201080.docx]

**Supplementary Data: ZINB with Complete Analysis**

**Table 1**

*Summary of results from the zero-inflated negative binomial model, all-cause ED visits without hospitalization*

| Parameter | ZINB | | | Prob Chi Sq |
| --- | --- | --- | --- | --- |
|  | RR | RR Lower | RR Upper |  |
| Traditional | 1.00 | 0.99 | 1.01 | 0.5899 |
| No enrolment | 1.14 | 1.12 | 1.17 | <0.0001 |
| Team based | 1 | 1 | 1 |  |
| Sex F | 0.94 | 0.93 | 0.95 | <0.0001 |
| Sex M | 1 | 1 | 1 |  |
| Patient age 50-64 | 0.87 | 0.86 | 0.89 | <0.0001 |
| Patient age 65-74 | 0.77 | 0.77 | 0.79 | <0.0001 |
| Patient age 75-84 | 0.82 | 0.80 | 0.83 | <0.0001 |
| Patient age 85+ | 0.85 | 0.83 | 0.87 | <0.0001 |
| Patient age 34-49 | 1 | 1 | 1 |  |
| ADG Total Score | 1.09 | 1.09 | 1.09 | <0.0001 |
| Asthma prevalence | 1.05 | 1.03 | 1.06 | <0.0001 |
| Income Quintile 2 | 0.90 | 0.89 | 0.91 | <0.0001 |
| Income Quintile 3 | 0.90 | 0.88 | 0.92 | <0.0001 |
| Income Quintile 4 | 0.85 | 0.84 | 0.87 | <0.0001 |
| Income Quintile 5 | 0.83 | 0.82 | 0.85 | <0.0001 |
| Income Quintile 1 | 1 | 1 | 1 |  |
| Rurality index 2008 | 1.01 | 1.01 | 1.01 | <0.0001 |
| Resource utilization band | 1.00 | 0.99 | 1.01 | 0.66 |
| ONMARG Summary Score | 1.09 | 1.08 | 1.10 | <0.0001 |
| Immigration 1 | 0.82 | 0.80 | 0.83 | <0.0001 |
| Immigration 0 | 1 | 1 | 1 |  |
| COCI missing | 0.76 | 0.72 | 0.81 | <0.0001 |
| COCI 0 – 0.20 | 2.42 | 2.38 | 2.46 | <0.0001 |
| COCI 0.21 – 0.30 | 1.63 | 1.60 | 1.65 | <0.0001 |
| COCI 0.31 – 0.50 | 1.34 | 1.31 | 1.36 | <0.0001 |
| COCI 0.51-1.0 | 1 | 1 | 1 | 1 |

Lagrange Multiplier Statistics: Chi-Square value of 3,554; Pr>ChiSq < 0.0001

Number of observations read: 55,994

Number of observations included in model: 39,058

Number of observations missing and replaced with zeros: 20,087

**Table 2**

*Summary of results from the zero-inflated negative binomial model, all-cause ED visits with hospitalization*

| Parameter | ZINB | | | Prob Chi Sq |
| --- | --- | --- | --- | --- |
|  | RR | RR Lower | RR Upper |  |
| Traditional | 1.00 | 0.97 | 1.02 | 0.671 |
| No enrolment | 1.11 | 1.07 | 1.15 | <0.0001 |
| Team based | 1 | 1 | 1 |  |
| Sex F | 0.96 | 0.94 | 0.98 | <0.0001 |
| Sex M | 1 | 1 | 1 |  |
| Patient age 50-64 | 1.10 | 1.06 | 1.13 | <0.0001 |
| Patient age 65-74 | 1.19 | 1.15 | 1.24 | <0.0001 |
| Patient age 75-84 | 1.34 | 1.29 | 1.39 | <0.0001 |
| Patient age 85+ | 1.53 | 1.46 | 1.59 | <0.0001 |
| Patient age 34-49 | 1 | 1 | 1 |  |
| ADG Total Score | 1.03 | 1.02 | 1.03 | <0.001 |
| Asthma prevalence | 0.94 | 0.92 | 0.96 | <0.001 |
| Income Quintile 2 | 0.95 | 0.92 | 0.97 | 0.0002 |
| Income Quintile 3 | 0.98 | 0.94 | 1.01 | 0.165 |
| Income Quintile 4 | 0.99 | 0.95 | 1.02 | 0.460 |
| Income Quintile 5 | 0.98 | 0.94 | 1.02 | 0.353 |
| Income Quintile 1 | 1 | 1 | 1 |  |
| Rurality index 2008 | 1.00 | 1.00 | 1.00 | <0.0001 |
| Resource utilization band | 1.07 | 1.06 | 1.09 | <0.0001 |
| ONMARG Summary Score | 1.07 | 1.05 | 1.1 | <0.0001 |
| Immigration 1 | 0.93 | 0.90 | 0.97 | 0.0002 |
| Immigration 0 | 1 | 1 | 1 |  |
| COCI missing | 1.80 | 1.45 | 2.24 | <0.0001 |
| COCI 0 – 0.20 | 2.38 | 2.30 | 2.47 | <0.0001 |
| COCI 0.21 – 0.30 | 1.52 | 1.46 | 1.59 | <0.0001 |
| COCI 0.31 – 0.50 | 1.25 | 1.20 | 1.30 | <0.0001 |
| COCI 0.51-1.0 | 1.06 | 1.01 | 1.1 | 1 |

Lagrange Multiplier Statistics: Chi-Square value of 1769.23; Pr>ChiSq < 0.0001

Number of observations read: 55,994

Number of observations included in model: 18,909

Number of observations missing and replaced with zero: 37,085

**Table 3**

*Summary of results from the zero-inflated negative binomial model, all -cause direct hospitalization*

| Parameter | ZINB | | | Prob Chi Sq |
| --- | --- | --- | --- | --- |
|  | RR | RR Lower | RR Upper |  |
| Traditional | 0.99 | 0.95 | 1.02 | 0.469 |
| No enrolment | 0.94 | 0.88 | 1.01 | 0.083 |
| Team based | 1 | 1 | 1 |  |
| Sex F | 0.95 | 0.92 | 0.98 | 0.002 |
| Sex M | 1 | 1 | 1 |  |
| Patient age 50-64 | 1.08 | 1.02 | 1.13 | 0.006 |
| Patient age 65-74 | 1.09 | 1.03 | 1.15 | 0.002 |
| Patient age 75-84 | 1.10 | 1.04 | 1.16 | 0.002 |
| Patient age 85+ | 1.16 | 1.06 | 1.28 | 0.002 |
| Patient age 34-49 | 1 | 1 | 1 |  |
| ADG Total Score | 1.01 | 1.00 | 1.01 | 0.494 |
| Asthma prevalence | 0.95 | 0.92 | 0.99 | 0.023 |
| Income Quintile 2 | 1.03 | 0.98 | 1.08 | 0.264 |
| Income Quintile 3 | 1.00 | 0.95 | 1.06 | 0.903 |
| Income Quintile 4 | 1.00 | 0.93 | 1.07 | 0.889 |
| Income Quintile 5 | 0.97 | 0.90 | 1.05 | 0.462 |
| Income Quintile 1 | 1 | 1 | 1 |  |
| Rurality index 2008 | 1 | 1 | 1 | 0.601 |
| Resource utilization band | 1.03 | 1.01 | 1.06 | 0.006 |
| ONMARG Summary Score | 1.00 | 0.96 | 1.04 | 0.925 |
| Immigration 1 | 0.98 | 0.92 | 1.04 | 0.509 |
| Immigration 0 | 1 | 1 | 1 |  |
| COCI missing | 1.08 | 0.87 | 1.34 | 0.461 |
| COCI 0 – 0.20 | 1.33 | 1.27 | 1.40 | <0.0001 |
| COCI 0.21 – 0.30 | 1.13 | 1.07 | 1.19 | <0.0001 |
| COCI 0.31 – 0.50 | 1.07 | 1.02 | 1.13 | 0.006 |
| COCI 0.51-1.0 | 1 | 1 | 1 |  |

Lagrange Multiplier Statistics: Chi-Square value of 1994.61; Pr>ChiSq < 0.0001

Number of observations read: 55,994

Number of observations included in model: 10,877

Number of observations missing and replaced with zeros: 45,117

**Table 4**

*Summary of results from the zero-inflated negative binomial model, all-cause 30-day readmissions*

| Parameter | ZINB | | | Prob Chi Sq |
| --- | --- | --- | --- | --- |
|  | RR | RR Lower | RR Upper |  |
| Traditional | 0.95 | 0.90 | 0.99 | 0.027 |
| No enrolment | 1.17 | 1.08 | 1.27 | <0.0001 |
| Team based | 1 | 1 | 1 |  |
| Sex F | 1.00 | 0.96 | 1.05 | 0.916 |
| Sex M | 1 | 1 | 1 |  |
| Patient age 50-64 | 0.92 | 0.85 | 0.99 | 0.026 |
| Patient age 65-74 | 0.84 | 0.78 | 0.91 | <0.0001 |
| Patient age 75-84 | 0.85 | 0.78 | 0.92 | <0.0001 |
| Patient age 85+ | 0.88 | 0.80 | 0.96 | 0.0066 |
| Patient age 34-49 | 1 | 1 | 1 |  |
| ADG Total Score | 1.02 | 1.01 | 1.03 | <0.001 |
| Asthma prevalence | 0.94 | 0.90 | 0.99 | 0.0279 |
| Income Quintile 2 | 0.98 | 0.82 | 1.05 | 0.587 |
| Income Quintile 3 | 1.03 | 0.96 | 1.11 | 0.403 |
| Income Quintile 4 | 1.10 | 1.01 | 1.20 | 0.346 |
| Income Quintile 5 | 1.06 | 0.96 | 1.18 | 0.213 |
| Income Quintile 1 | 1 | 1 | 1 |  |
| Rurality index 2008 | 1 | 1 | 1 | 0.8445 |
| Resource utilization band | 1.03 | 0.99 | 1.06 | 0.1204 |
| ONMARG Summary Score | 1.11 | 1.06 | 1.16 | <0.0001 |
| Immigration 1 | 0.99 | 0.91 | 1.07 | 0.729 |
| Immigration 0 | 1 | 1 | 1 |  |
| COCI missing | 1.02 | 0.38 | 2.73 | 0.973 |
| COCI 0 – 0.20 | 1.90 | 1.72 | 2.11 | <0.0001 |
| COCI 0.21 – 0.30 | 1.35 | 1.21 | 1.51 | <0.0001 |
| COCI 0.31 – 0.50 | 1.18 | 1.05 | 1.33 | 0.0049 |
| COCI 0.51-1.0 | 1 | 1 | 1 | 1 |

Lagrange Multiplier Statistics: Chi-Square value of 235. 39; Pr>ChiSq < 0.0001

Number of observations read: 55,994

Number of observations included in model: 4,882

Number of observations missing and replaced with zeros: 51,112

**Table 5**

*Summary of results from the zero-inflated negative binomial model, COPD-related ED visits without hospitalization*

| Parameter | ZINB | | | Prob Chi Sq |
| --- | --- | --- | --- | --- |
|  | RR | RR Lower | RR Upper |  |
| Traditional | 1.08 | 1.01 | 1.16 | 0.023 |
| No enrolment | 1.75 | 1.59 | 1.93 | <0.0001 |
| Team based | 1 | 1 | 1 |  |
| Sex F | 0.88 | 0.83 | 0.94 | <0.0001 |
| Sex M | 1 | 1 | 1 |  |
| Patient age 50-64 | 1.20 | 1.09 | 1.32 | 0.0002 |
| Patient age 65-74 | 1.05 | 0.95 | 1.17 | 0.343 |
| Patient age 75-84 | 0.87 | 0.77 | 0.98 | 0.0273 |
| Patient age 85+ | 0.82 | 0.68 | 0.99 | 0.0394 |
| Patient age 34-49 | 1 | 1 | 1 |  |
| ADG Total Score | 1.01 | 1.00 | 1.02 | 0.172 |
| Asthma prevalence | 1.00 | 0.93 | 1.07 | 0.932 |
| Income Quintile 2 | 0.95 | 0.87 | 1.04 | 0.241 |
| Income Quintile 3 | 0.91 | 0.91 | 1.01 | 0.0775 |
| Income Quintile 4 | 0.91 | 0.80 | 1.04 | 0.1551 |
| Income Quintile 5 | 0.95 | 0.82 | 1.10 | 0.504 |
| Income Quintile 1 | 1 | 1 | 1 |  |
| Rurality index 2008 | 1.01 | 100 | 1.01 | <0.0001 |
| Resource utilization band | 1.02 | 0.97 | 1.06 | 0.430 |
| ONMARG Summary Score | 1.20 | 1.12 | 1.27 | <0.0001 |
| Immigration 1 | 0.68 | 0.56 | 0.82 | <0.0001 |
| Immigration 0 | 1 | 1 | 1 |  |
| COCI missing | 1.20 | 0.62 | 2.32 | 0.595 |
| COCI 0 – 0.20 | 3.43 | 2.93 | 4.02 | <0.0001 |
| COCI 0.21 – 0.30 | 2.07 | 1.75 | 2.46 | <0.0001 |
| COCI 0.31 – 0.50 | 1.65 | 1.38 | 1.96 | <0.0001 |
| COCI 0.51-1.0 | 1 | 1 | 1 | 1 |

Lagrange Multiplier Statistics: Chi-Square value of 246.97; Pr>ChiSq < 0.0001

Number of observations read: 55,994

Number of observations included in model: 39,058

Number of observations missing and replaced with zeros: 16,936

**Table 6**

*Summary of results from the zero-inflated negative binomial Poisson model, COPD-related ED visits with hospitalization*

| Parameter | ZINB | | | Prob Chi Sq |
| --- | --- | --- | --- | --- |
|  | RR | RR Lower | RR Upper |  |
| Traditional | 0.97 | 0.89 | 1.06 | 0.510 |
| No enrolment | 1.17 | 1.01 | 1.34 | 0.0311 |
| Team based | 1 | 1 | 1 |  |
| Sex F | 1.01 | 0.93 | 1.09 | 0.791 |
| Sex M | 1 | 1 | 1 |  |
| Patient age 50-64 | 0.86 | 0.74 | 1.00 | 0.043 |
| Patient age 65-74 | 0.73 | 0.62 | 0.85 | <0.0001 |
| Patient age 75-84 | 0.72 | 0.61 | 0.84 | <0.0001 |
| Patient age 85+ | 0.77 | 0.64 | 0.92 | 0.0050 |
| Patient age 34-49 | 1 | 1 | 1 |  |
| ADG Total Score | 1.00 | 0.99 | 1.02 | 0.852 |
| Asthma prevalence | 0.90 | 0.82 | 0.99 | 0.024 |
| Income Quintile 2 | 0.81 | 0.72 | 0.90 | 0.0002 |
| Income Quintile 3 | 0.75 | 0.65 | 0.86 | <0.0001 |
| Income Quintile 4 | 0.79 | 0.67 | 0.93 | 0.0056 |
| Income Quintile 5 | 0.82 | 0.68 | 0.99 | 0.0381 |
| Income Quintile 1 | 1 | 1 | 1 |  |
| Rurality index 2008 | 1 | 0.99 | 1 | 0.0034 |
| Resource utilization band | 0.98 | 0.93 | 1.03 | 0.3456 |
| ONMARG Summary Score | 1.00 | 0.92 | 1.09 | 0.9556 |
| Immigration 1 | 1.02 | 0.85 | 1.23 | 0.8016 |
| Immigration 0 | 1 | 1 | 1 |  |
| COCI missing | 2.68 | 0.03 | 286.63 | 0.6652 |
| COCI 0 – 0.20 | 4.23 | 3.09 | 5.77 | <0.0001 |
| COCI 0.21 – 0.30 | 2.13 | 1.54 | 2.96 | <0.0001 |
| COCI 0.31 – 0.50 | 1.55 | 1.11 | 2.18 | 0.0112 |
| COCI 0.51-1.0 | 1 | 1 | 1 | 1 |

Lagrange Multiplier Statistics: Chi-Square value of 201. 40; Pr>ChiSq < 0.0001

Number of observations read: 55,994

Number of observations included in model: 18,909

Number of observations missing and replaced with zeros: 37,085

**Table 7**

*Summary of results from the zero-inflated negative binomial model, COPD-related direct hospitalization*

| Parameter | ZINB | | | Prob Chi Sq |
| --- | --- | --- | --- | --- |
|  | RR | RR Lower | RR Upper |  |
| Traditional | 1.16 | 0.88 | 1.5 | 0.2905 |
| No enrolment | 0.53 | 0.35 | 0.81 | 0.0032 |
| Team based | 1 | 1 | 1 |  |
| Sex F | 0.83 | 0.64 | 1.09 | 0.1919 |
| Sex M | 1 | 1 | 1 |  |
| Patient age 50-64 | 1.22 | 0.55 | 2.72 | 0.6222 |
| Patient age 65-74 | 1.93 | 0.84 | 4.46 | 0.1238 |
| Patient age 75-84 | 1.42 | 0.63 | 3.22 | 0.3991 |
| Patient age 85+ | 4.62 | 2.03 | 10.50 | 0.0003 |
| Patient age 34-49 | 1 | 1 | 1 |  |
| ADG Total Score | 1.02 | 0.98 | 1.06 | 0.3239 |
| Asthma prevalence | 0.94 | 0.72 | 1.23 | 0.6429 |
| Income Quintile 2 | 0.97 | 0.71 | 1.32 | 0.836 |
| Income Quintile 3 | 1.10 | 0.77 | 1.57 | 0.5844 |
| Income Quintile 4 | 0.97 | 0.59 | 1.59 | 0.9023 |
| Income Quintile 5 | 0.70 | 0.41 | 1.18 | 0.1749 |
| Income Quintile 1 | 1 | 1 | 1 |  |
| Rurality index 2008 | 1.00 | 1.00 | 1.01 | 0.110 |
| Resource utilization band | 0.89 | 0.78 | 1.01 | 0.074 |
| ONMARG Summary Score | 0.98 | 0.78 | 1.22 | 0.842 |
| Immigration 1 | 0.46 | 0.27 | 0.79 | 0.004 |
| Immigration 0 | 1 | 1 | 1 |  |
| COCI missing | 0.86 | 0.33 | 2.24 | 0.7505 |
| COCI 0 – 0.20 | 2.74 | 1.89 | 3.97 | <0.0001 |
| COCI 0.21 – 0.30 | 1.88 | 1.18 | 2.99 | 0.0078 |
| COCI 0.31 – 0.50 | 2.40 | 1.58 | 3.66 | <0.0001 |
| COCI 0.51-1.0 | 1 | 1 | 1 | 1 |

Lagrange Multiplier Statistics: Chi-Square value of 4.87 Pr>ChiSq 0.014

Number of observations read: 55,994

Number of observations included in model:10,877

Number of observations missing and replaced with zeros: 45,117

**Table 8**

*Summary of results from the zero-inflated negative binomial model, COPD-related 30-day readmission*

| Parameter | ZINB | | | Prob Chi Sq |
| --- | --- | --- | --- | --- |
|  | RR | RR Lower | RR Upper |  |
| Traditional | 0.91 | 0.79 | 1.06 | 0.238 |
| No enrolment | 2.26 | 1.86 | 2.75 | <0.0001 |
| Team based | 1 | 1 | 1 |  |
| Sex F | 1.23 | 1.08 | 1.40 | 0.0022 |
| Sex M | 1 | 1 | 1 |  |
| Patient age 50-64 | 0.65 | 0.52 | 0.82 | 0.0002 |
| Patient age 65-74 | 0.34 | 0.26 | 0.44 | <0.0001 |
| Patient age 75-84 | 0.38 | 0.30 | 0.48 | <0.0001 |
| Patient age 85+ | 0.40 | 0.29 | 0.55 | <0.0001 |
| Patient age 34-49 | 1 | 1 | 1 |  |
| ADG Total Score | 0.97 | 0.95 | 0.99 | 0.0167 |
| Asthma prevalence | 0.78 | 0.66 | 0.92 | 0.0025 |
| Income Quintile 2 | 0.90 | 0.74 | 1.09 | 0.267 |
| Income Quintile 3 | 0.85 | 0.67 | 1.08 | 0.1856 |
| Income Quintile 4 | 1.34 | 1.02 | 1.76 | 0.0381 |
| Income Quintile 5 | 1.42 | 1.03 | 1.96 | 0.0327 |
| Income Quintile 1 | 1 | 1 | 1 |  |
| Rurality index 2008 | 1 | 0.99 | 1.00 | 0.2441 |
| Resource utilization band | 1.08 | 0.99 | 1.17 | 0.0789 |
| ONMARG Summary Score | 1.36 | 1.19 | 1.56 | <0.0001 |
| Immigration 1 | 0.71 | 0.50 | 1.01 | 0.0539 |
| Immigration 0 | 1 | 1 | 1 |  |
| COCI missing | 1.80 | 0.14 | 23.29 | 0.6520 |
| COCI 0 – 0.20 | 2.95 | 1.61 | 5.37 | 0.0004 |
| COCI 0.21 – 0.30 | 1.70 | 0.91 | 3.16 | 0.0979 |
| COCI 0.31 – 0.50 | 1.02 | 0.53 | 1.98 | 0.9574 |
| COCI 0.51-1.0 | 1 | 1 | 1 | 1 |

Lagrange Multiplier Statistics: Chi-Square value of 85; Pr>ChiSq < 0.0001

Number of observations read: 55,994

Number of observations included in model: 4,882

Number of observations missing and replaced with zeros: 54,263
